# Accelerated aging in perinatally HIV-infected children: clinical manifestations and pathogenetic mechanisms

**DOI:** 10.18632/aging.101622

**Published:** 2018-11-11

**Authors:** Elena Chiappini, Martina Bianconi, Annalisa Dalzini, Maria Raffaella Petrara, Luisa Galli, Carlo Giaquinto, Anita De Rossi

**Affiliations:** 1 Infectious Disease Unit, Meyer Children’s Hospital, Department of Science Health, University of Florence, Florence, Italy; 2 Section of Oncology and Immunology, Department of Surgery, Oncology and Gastroenterology, Unit of viral Oncology and AIDS Reference Center, University of Padova, Padova, Italy; 3 Department of Mother and Child Health, University of Padova, Padova, Italy; 4 Istituto Oncologico Veneto IOV-IRCCS, Padova, Italy

**Keywords:** HIV, children, aging, telomeres, antiretroviral therapy

## Abstract

Background: Premature aging and related diseases have been documented in HIV-infected adults. Data are now emerging also regarding accelerated aging process in HIV-infected children.

Methods: A narrative review was performed searching studies on PubMed published in English language in 2004-2017, using appropriate key words, including “aging”, “children”, “HIV”, “AIDS”, “immunosenescence”, “pathogenesis”, “clinical conditions”.

Results: Premature immunosenescence phenotype of B and T cells in HIV-infected children is mediated through immune system activation and chronic inflammation. Ongoing inflammation processes have been documented by increased levels of pathogen-associated molecular patterns (PAMPS), increased mitochondrial damage, higher levels of pro-inflammatory cytokines, and a positive correlation between sCD14 levels and percentages of activated CD8^+^ cells. Other reported features of premature aging include cellular replicative senescence, linked to an accelerated telomeres shortening. Finally, acceleration of age-associated methylation pattern and other epigenetic modifications have been described in HIV-infected children. All these features may favor the clinical manifestations related to premature aging. Lipid and bone metabolism, cancers, cardiovascular, renal, and neurological systems should be carefully monitored, particularly in children with detectable viremia and/or with CD4/CD8 ratio inversion.

Conclusion: Aging processes in children with HIV infection impact their quality and length of life. Further studies regarding the mechanisms involved in premature aging are needed to search for potential targets of treatment.

## Introduction

Subsequently to the introduction of the combined highly active antiretroviral therapy (ART), life expectancy of HIV-infected adults has increased dramatically, but it is currently not yet comparable to that of healthy individuals [1]. Indeed, despite ART, the lifespan of HIV-infected individuals in Western countries is shortened by an average of 10 years [2], and accelerate aging processes and occurrence of precocious diseases have been reported in comparison to age-matched HIV-uninfected controls [3]. Aging is defined as a progressive loss of physiological integrity, with heterogeneous organ decline, naturally ending by death [4]. This process is associated with decreased ability to face stress, increased frailty, and increased prevalence of age-related comorbidities [4,5]. Accumulating data suggest that aging process does not spare HIV-infected children, as well as adults. It is important to underline that by the end of 2013, 3.3 million children under 15 years old were living with HIV infection worldwide, and 630,000 of them had access to ART [6]. These children receive antiretroviral drugs for all their lifetime and, having a longer life expectancy than in the past, must face a chronic condition. Pathogenic mechanisms of premature aging, that are well documented in HIV-infected adults, are now emerging also in HIV-infected children, with an impact on their quality and length of life [7–11]. Clearly, HIV infection has different characteristics in adults than in children. Perinatally infected children have higher HIV plasma viremia and faster disease progression compared to adults [12,13]. This slower control of viral replication may depend from the fact that the immune system is still maturing. Exposure to HIV or to ART since, or before, birth may affect premature aging and immune senescence in children, even more than in adults. This narrative review describes the pathogenic mechanisms of premature aging in children, possibly underlying the main related clinical features.

## RESULTS

### Clinical conditions related to premature aging in HIV-infected children

The introduction of ART has changed the natural history of pediatric HIV infection and mortality in children has decreased by over 80-90% in Europe [14,15]. Similar data has been reported in the United States where mortality rate in HIV-infected children declined from 7.2 per 100 child/year in 1994 to 0.6 per 100 child/year in 2006 [16]. Thus, HIV infection is now considered a chronic disease which persists for many decades. However, it has been estimated that HIV-infected children display mortality rates 30 times higher than uninfected children due to chronic diseases, such as metabolic, cardiovascular, kidney and neurological disorders, and cancers [7–11]. These “non-AIDS related pathologies” represent a group of conditions possibly associated with HIV-mediated aging; the ongoing inflammatory immune process, that may persist despite ART, may also drive the premature cellular aging. The whole picture is complex and probably due to the interaction of multiple biologic and pharmacologic mechanisms (Table 1).

**Table 1:** Clinical conditions related to premature aging in HIV-infected children.

**Features**	**Characteristics/mechanisms**	**References**	
**Renal function**	Synergy between VEGF-A, FGF-2 and the HIV Tat protein affect the structure of renal endothelial cells and podocytes, leading to a precocious renal disease.	Das Jr et al, 2016 Li J et al, 2017	[24] [25]
	Transmembrane TNF-α facilitates HIV infection of podocytes and renal endothelial cells		
	In children with HIV-related nephropathy, podocytes express TNF-α mRNA and protein, as described in other renal inflammatory diseases		
**Neuropsycological conditions**	Neuropsycological disorders despite effective ART are reported in HIV infected children (CNS is a reservoir for HIV replication, some drugs have poor CNS penetration, persistent immune activation is ongoing)	Cohen S et al, 2015 Vreeman RC et al, 2015 Wilmshurst JM et al, 2018 Van Arnhem LA et al, 2013	[29] [30] [31] [32]
	White matter signal abnormalities have been described in HIV-infected children on early ART	Ackermann et al, 2014	[33]
	Cerebrovascular disease has been reported in HIV infected children in the HAART era possibly due to inflammatory or autoimmune response against vascular wall	Connor MD et al, 2009 Hammond CK et al, 2016	[34] [35]
**Bone metabolism alterations**	Senescent phenotype of osteoblasts has been described in HIV- infected children	Warriner AH et al, 2014	[38]
	Precocious bone abnormalities may be related to HIV-driven chronic inflammation: IL-1, IL-6, IL-17, TNF-α boost osteoclast, suppress osteoblast activity and cause apoptosis	Masky KC et al, 2010 Puthanakit T et al, 2013 Gibellini D et al, 2008	[39] [40] [42]
	HIV Tat and Nef directly alter osteoblastic differentiation; HIV gp 120 promotes apoptosis of osteoblasts by upregulating TNF-α	Gibellini D et al, 2008 Beaupere C et al, 2015	[42] [43]
	HIV induces increased RANKL expression, stimulating osteoclastogenesis, and bone reabsorption	Natsag J et al, 2016	[41]
	High percentages of activated and senescent CD4^+^ and CD8^+^ T cells correlate with low bone mineral density	Manavalan JS et al, 2016	[44]
**Cardiovascular disease**	Coronary plaque is associated with markers of T-cell activation and E-selectine / endothelial inflammation / in HIV infected children	Mattingly AS et al , 2017	[51]
	Subclinical atherosclerosis is related with low CD8^+^ count	Sainz T et al, 2014	[50]
	Carotid intima-media thickness is related to high sensitivity C reactive protein levels	Ross AC et al, 2010	[52]
	ART-related lipodystrophy, dyslipidemia, and glucose intolerance predispose HIV-infected children to early cardiovascular disease	Loomba-Albrecht LA et al, 2014	[53]
**Endocrine alterations**	HIV-driven chronic inflammation can cause hypothalamic-pituitary-adrenal axis alterations and increasing glucorticoid production	Loomba-Albrecht LA et al,2014	[53]
	Lypodystrophy and dysplidemia have been found in HIV-infected children not receiving ART		
**Cancer risk**	Increased incidence of non-AIDS related malignances has been found HIV infected children, despite ART	Chiappini E et al et al, 2007 Alvaro-Meca A et al, 2011 Davidson A et al, 2011 Franceschi S et al, 2010	[54] [55] [56] [58]
	Chronic activation, increased cell turnover and accelerated immune senescence is involved in cancer development	Simard EP et al 2012 Chiappini E et al, 2014	[57] [10]

### *Renal function*

Renal function of HIV-infected children may be impaired not only due to the classic HIV-associated nephropathy (HIVAN), commonly reported in the pre-ART era, but also for the development of hemolytic uremic syndrome, thrombotic thrombocytopenic purpura, acute kidney injury, renal nephrotoxicity syndromes associated with some specific antiretroviral drugs (i.e. tenofovir), and for the ongoing inflammatory process. Overall, these conditions lead to a premature loss of renal function [17–19].

Compared to HIV-uninfected controls, the prevalence of albuminuria has been reported to be 2-5-fold higher in HIV-infected children [20]. Main risk factors are family history of renal disease, genetic predisposition (such as Apoliprotein (APOL)-1 renal risk variants), immune suppression, history of proteinuria, diabetes, hepatitis C virus co-infection, and treatment with certain antiretroviral drugs [20,21]. ART has dramatically reduced the incidence of HIVAN, but a clear benefit in non-HIVAN kidney disease has not been demonstrated [22]. APOL-1 renal risk variants are strongly associated with chronic kidney disease and especially with HIVAN in individuals with sub-Saharan African ancestry; about 18% of children with perinatal HIV infection and high risk APOL-1 genotype develops chronic kidney diseases, but biological reasons for this phenomenon are still unknown [23]. It has been demonstrated that synergy between Vascular Endothelial Cell Growth Factor (VEGF)-A, Fibroblast Growth Factor (FGF)-2 and the HIV Tat protein can affect *in vitro* cytoskeletal structure and permeability of cultured renal endothelial cells (REC) and podocytes, which compound the glomerular filtration barrier. Urine samples from HIV-infected children with renal diseases showed high levels of VEGF-A and FGF-2, and induced similar changes in cultured REC and podocytes [24]. In addition, a recent study has demonstrated that transmembrane TNF-α facilitates HIV infection *in vitro* of podocytes and REC of children with HIVAN [25]. These mechanisms may lead to a precocious renal disease in HIV-infected children.

### *Neuropsychological conditions*

Before the introduction of ART, HIV-infected children were often affected by HIV encephalopathy characterized by impaired brain growth, motor deficits and developmental delay [26]. After the introduction of ART, HIV encephalopathy has declined from 30%-50% to <2%, but other neuropsychological disorders have been reported at higher rates than in HIV-uninfected sex and age matched-controls with same ethnicity and socioeconomic status [27]. In addition, lower total intelligence quotient, language impairment, poorer working memory, gross and fine motor functioning and visual-motor impairment have been extensively reported in ART-treated HIV-infected children, more frequently than in healthy controls [27–30]. On the other hand, memory and executive functioning domains seem to be only slightly affected in HIV-infected children, while they are frequently compromised in HIV-infected adults [29,30]. This discrepancy in neurological impairment between children and adults may be due to the fact that in adults HIV neuro invasion occurs long after neuronal development has ended, while in children the viral spread is parallel to the neurological development [31]. Magnetic resonance imaging studies demonstrated that the neurological abnormalities observed in children are associated with ventricular and/or sulcal enlargement and white matter lesions. These findings are reported both in HIV-infected ART-treated and non-treated children, suggesting that the starting phase of active viral replication in central nervous system (CNS), occurring prenatally and prior to treatment, is crucial for the development of neuro-imaging abnormalities [32,33]. One study demonstrated white matter abnormalities in children under 3 years of age that had started ART before 3 months of age, indicating that infiltration and damage of the CNS by HIV occurs at an early stage of infection [33]. In addition, CNS is a reservoir for HIV replication that causes itself immune activation and precocious neurological damage, despite ART [32]. The role of long-term ART is also unclear: despite good immunologic and systemic viral control, cognitive impairment may persist, probably due to the poor drug CNS penetration, or persistent immune activation despite ART. On the other hand, molecules with high CNS penetration may also display neurotoxic effect [30]. Another typical neurological finding in HIV-infected children is the HIV-associated cerebral vasculopathy, defined as an arteriopathy of medium-sized cerebral vessels with radiological evidence of vessel stenosis, occlusion or aneurysmal dilatation; it can be asymptomatic, or cause stroke, encephalopathy, or cognitive impairment and its prevalence does not decline after ART introduction [34,35]. The pathogenesis of such a cerebrovascular disease is still unclear, but it may be due to the inflammatory or autoimmune response against vascular wall [35].

### *Bone metabolism alterations*

Children with HIV infection can develop precocious bone abnormalities with an increased risk of osteoporosis and fractures [36]. The risk of bone disorder is greater in children than in adults, because bone mass increases during childhood, accelerating during adolescence [37,38]. The cause is multifactorial and not fully clear, but many demographic, genetic, hormonal, nutritional factors, HIV levels and drugs are involved. Long-term ART, especially including tenofovir, disoproxil, and fumarate, is associated with greater bone loss [36,37]. Furthermore, precocious bone abnormalities are probably correlated with HIV-related chronic inflammation: pro-inflammatory cytokines, such as interleukin (IL)-1, IL-6, IL-17, and TNF-α can boost osteoclast and suppress osteoblast activity or cause their apoptosis [39–41]. HIV infection also induces an increased expression of receptor activator of nuclear factor (NF)-kB ligand (RANKL), stimulating osteoclastogenesis, and subsequent bone remodeling and reabsorption [41]. In addition, lower bone mineral density is correlated with a senescent phenotype of the osteoblast [38]. Several studies have demonstrated that HIV gp120 glycoprotein promotes apoptosis of osteoblasts through an up-regulation of TNF-α, and HIV proteins Tat and Nef induce precocious aging in bone marrow mesenchymal stem cells by increasing inflammation and autophagy processes [42,43]. Manavalan et al. demonstrated that higher percentages of activated and senescent CD4^+^ and CD8^+^ T cells correlated with lower numbers of circulating osteoblastic precursor and lower bone mineral density (BMD) in perinatally HIV-infected children and adolescents [44]. However, Jimenez et al. showed that the low nadir CD4^+^ T cell, but not markers of T-cell activation or senescence, was an independent predictor for low BMD [45]. Thus, further and larger studies investigating the correlation between immunoactivation, immunosenescence and bone mass in HIV-infected children are needed.

### *Cardiovascular disease*

After the introduction of ART, cardiovascular risk and incidence of related cardiovascular complications (i.e. cardiomyopathy) decreased significantly. However, asymptomatic structural abnormalities in HIV-infected children persist during ART and they may be related to subsequent clinically evident diseases in adult life [46]. In HIV-infected adults, many factors may contribute to vascular diseases, including classical risk factors (i.e., obesity, diabetes, hypertension, sedentary life, smoke), the side effects of long-term ART, and HIV-related inflammation and immune activation on heart and vessels [47–49]. The carotid intima-media thickness is considered as a reliable marker of subclinical atherosclerosis and consequently of cardiovascular disease) [49,50]. Sainz et al. demonstrated that HIV infection in children is associated with thicker carotid intima media, and that a low CD4^+^ T-cell nadir is related to an increased carotid intima thickness; however, no relation was found between increased carotid intima thickness and inflammation, immune activation, or senescence [50]. On the other hand, a recent study in HIV-infected children has shown that coronary plaque was positively associated with activated CD8^+^ cells and levels of E-selectin, a marker of endothelial inflammation; these data support that immune activation and endothelial inflammation may accelerate the early stages of atherosclerosis [51]. In a small study, higher levels of high sensitivity C-reactive protein was found in HIV- infected children compared to healthy controls, which support a role for inflammation in cardiovascular risk in this population [52].

### *Endocrine alterations*

Chronic HIV infection drives the release of proinflammatory cytokines, such as IL-1, IL-6, TNF-α, and interferon (IFN) type 1, that can cause hypothalamic-pituitary-adrenal axis alteration, increasing glucocorticoid production [53]. In addition, HIV infected children may show changes in lipid and glucose metabolism including lipodystrophy, dyslipidemia, and glucose intolerance that predispose to early cardiovascular disease. The main cause of these alterations seems to be the antiretroviral therapy, but a role of HIV infection itself cannot be excluded because lipodystrophy and dyslipidemia has been described in HIV-infected children ART-naïve [53].

### *Cancer risk*

HIV-infected children display an increased cancer risk. Since ART introduction decreased rates of the three AIDS defining malignancies (ADM), i.e. Kaposi sarcoma, non-Hodgkin lymphoma (NHL) and cervical cancer, have been reported in children [54]. However, the incidence of several other “non-ADM” is increasing [10]. One study highlighted that, comparing the periods 1997-1999 and 2003-2008, ADM diagnoses rate fell from 9.1 to 1.0 cancers per 1000 children/year, but in the same periods non-ADM diagnoses rate rose from 0.6 to 8.7 cancers per 1000 children/year [55]. Typical non-ADM are anal cancer, Hodgkin’s disease, leiomyosarcoma, squamous conjunctival carcinoma and hepatocarcinoma [10]. In addition, non-ADM present atypical histological subtypes and unusual sites compared to those occurring in immunocompetent children [56]. ADM are mainly caused by immunosuppression and co-infection with oncogenic viruses, such as HHV8, EBV, HPV [10]. The pathogenesis of non-ADM involves chronic immune activation, increased cell turnover and accelerated immune senescence (see below), and their incidence is rising [57,58].

### Pathogenetic mechanisms of premature aging in HIV-infected children

The effects of HIV-infection in children and adolescents, especially those who were perinatally infected, and thus are dealing with the virus from birth, are complex. HIV plasma viremia is higher in children than in adults, and the disease progresses faster [13] due to the incomplete maturation of their immune system. Indeed, infants’ immune systems are more plastic and dynamic than adults, and deserve particular attention. The reasons for the differences between children and adults are yet to be fully cleared, but one contributing factor may be the much higher thymic output of T-cells in children than in adults [59]. The mechanisms of premature aging and related disease in HIV-infected children compared to HIV-infected adults is therefore affected by the age-related changes, and most likely result from both lifelong exposure to pathogens and antigens, as well as intrinsic changes in immune cells [60].

The pathogenic mechanisms of aging, and in particular the accelerated immunosenescence, have been partly described in adults, whereas few studies are available in children.

### *Immunosenescence profile and aging*

Results from several studies suggest that peripheral blood lymphocytes of perinatally HIV-infected children have typical features of an aging immune system. The following features have been considered as hallmarks of the aging process: i) high percentage of activated CD45RO^+^CD95^+^ T cells, which lack the costimulatory CD28 molecule and are prone to undergo apoptosis, ii) increased levels of Natural Killer (NK) cells, iii) decrease of CD19^+^ B lymphocytes and iv) mitochondrial damage (Table 2). Although the CD4^+^ T cell is the target of HIV infection, also CD8^+^ T cell compartment was found to be largely impaired in HIV-infected children, as well as in adults. Mansoor et al. studied T cell subsets over the first year of life of HIV-infected ART-untreated children, HIV-exposed uninfected children (i.e. born to HIV-infected mothers) and HIV-unexposed children; they found that in HIV-infected children the naïve CD8^+^CD45RA^+^CCR7^+^ T cells were significantly decreased, while the percentage of CD8^+^CD45RA^-^CCR7^-^ effector memory cells and terminally differentiated CD8^+^CD45RA^+^CCR7^-^ cells were increased compared to the control cohorts: this profile may reflect the immune activation that drives cells into the state of terminal differentiation [61]. Notably, HIV-infected children over one year of age had also a significant higher percentage of CD8^+^ cells expressing the CD57^+^ molecule, a marker of replicative senescence [62,63], than controls [61]. Similar data were observed by Diaz et al., that studied the immunosenescence of CD8^+^ T-cell subsets in perinatally HIV-infected children, and found that these children had a higher percentage of senescent CD8^+^CD57^+^ cells than age-matched healthy children, but, differently from the previous study, only HIV-infected children with detectable viral load showed increased frequencies of effector memory and terminally differentiated T cells [64]. Notably, the alteration of memory and senescent T cells in infants may also have implications on the efficacy of childhood vaccination [61] and on cancers development; the progressive increase of senescent T cells with a senescent-associated secretory phenotype (SASP) can hamper immune surveillance during antigenic presentation facilitating the development of tumors [65, 66]. Another study suggested a correlation between the persistence of inverted CD4/CD8 ratio during ART and the premature immunosenescence in HIV-infected children [67]. Notably, inversion of the CD4/CD8 ratio (<1) is a hallmark of untreated HIV infection, but in some cases this alteration persists despite effective ART and viral suppression, and is associated with increased levels of activated and senescent T cells, and a skewed T-cell phenotype from naıve toward effector memory [67]. Increased levels of T cells prone to apoptosis along with increased levels of NK cells and mitochondrial damage has been also reported in one study [68].

**Table 2:** Pathogenetic mechanisms of premature aging in HIV-infected children.

**Features**	**Characteristics/mechanisms**	**References**	
**T cell profile**	Increased activated CD45RO^+^CD95^+^ T cells	Sainz T et al, 2013	[67]
	Decreased naïve CD8^+^CD45RA^+^CCR7^+^ cells	Mansoor N et al, 2009 Sainz T et al, 2013	[61] [67]
	Increased CD8^+^CD45RA^-^CCR7^-^ effector memory		
	Increased CD8^+^CD45RA^+^CCR7^-^ terminally differentiated cells	Mansoor N et al, 2009	[61]
	Increased of CD8^+^CD28^-^CD57^+^ senescent cells	Mansoor N et al, 2009 Diaz L et al, 2012 Gianesin K et al, 2016	[61] [64] [86]
	Increased CD8^+^CD38^+^HLA-DR^+^ activated cells	Sainz T et al, 2013 Gianesin K et al, 2016	[67] [86]
	Increased PD-1^+^ exhausted cells	Gianesin K et al, 2016	[86]
	Inverted CD4/CD8 ratio	Sainz T et al, 2013	[67]
**B cell profile**	Impaired immune response to vaccines	Hart M et al, 2007 Moir S et al, 2008 Cagigi A et al, 2014 Siberry GK et al, 2015	[69] [72] [71] [70]
	Increased levels of immature transitional B cells	Moir S et al, 2009	[73]
	Increased levels of activated memory B cells	Moir S et al, 2009 Cagigi A et al, 2014	[73] [71]
	Increased levels of double negative B cells [CD27^-^IgD^-^)	Moir S et al, 2009 Cagigi A et al, 2014 Rinaldi et al, 2017	[73] [71] [74]
**NK cell profile**	Increased levels of NK cells	Viganò A et al, 2001	[68]
**Inflammation**	Increased levels of PAMPs [sCD14 and LPS) and pro-inflammatory cytokines	Marks M et al , 2013 Gianesin et al, 2016	[94] [86]
	Correlation between sCD14 and percentages of activated CD8^+^ cells	Gianesin et al, 2016	[86]
	Increased mitochondrial damage	Viganò A et al, 2001	[68]
**Replicative cell senescence**	Telomere shortening	Côté HC et al, 2012 Gianesin K et al, 2016	[85] [86]
	NRTIs inhibition of TERT, leading to premature telomeres shortening	Liu X et al, 2007 Tressler R et al, 2012 Hukezalie KR et al, 2012	[83] [82] [84]
	Downmodulation of telomerase expression and activity by HIV Tat protein	Ballon G et al, 2001 Reynoso R et al, 2006 Franzese O et al, 2007	[89] [90] [91]
**Epigenetic changes**	CpG DNA methylation	Gross AM et al, 2016	[99]
	Acceleration of age-associated methylation pattern	Rickabaugh TM et al,2015	[100]

Several studies suggested that HIV infection can also affect B cell function in both adults and children; these alterations can, at least in part, persist during ART and can impair immune response to vaccines and increase susceptibility to vaccine-preventable diseases [69–72]. The main B cells alterations observed in HIV-infected subjects include increased levels of immature transitional B cells, activated memory double negative B cells (CD27^-^IgD^-^), and decreased resting memory B cells subset [73]. Cagigi et al. demonstrated that HIV-infected children with undetectable viral load showed B cell alterations typical of elderly people, such as an increased number of mature-activated and double negative B cells, and these findings have been associated with a poor humoral response versus seasonal influenza vaccination [71]. Rinaldi et al. investigated antibody responses versus flu vaccination in different groups of subjects on the basis of their HIV status and age: young people with HIV infection on ART showed increased frequencies of double negative B cells and decreased plasmablasts similar to older healthy controls, supporting that despite ART, HIV infection drives precocious immunosenescence of B cells [74].

### *Cellular replicative senescence and aging*

An important mechanism of aging and immunosenescence involves telomeres. Telomeres are repetitive DNA sequences at the end of chromosomes and are essential for protecting chromosome integrity [75]. Telomeres are progressively shortened during each cell division due to end-replication problems of DNA polymerase; when a critical length is reached the cell undergoes cycle arrest and replicative senescence. Senescent cells have a SASP phenotype and secrete factors that can influence age-associated diseases [76]. During life, telomeres get shorter with increasing age, infections, oxidative damage and other factors [77]. Premature telomere shortening leads to premature aging, and this shortening has been correlated with the development of particular pathologies, such as cardiovascular disease and cancer [78,79]. The pathogenic mechanism(s) underlying the accelerated telomere shortening in HIV-infected children is still poorly understood. Telomerase, a ribonucleoprotein complex containing an internal RNA component (TR or TERC) and a catalytic protein (TERT, Telomerase Reverse Transcriptase), enables telomere elongation; it is active in cancer cells and, transiently, in tissue in rapid proliferation [75]. HIV reverse transcriptase shares homology with TERT [80,81]; thus NRTIs, such as zidovudine and abacavir, may inhibit TERT and consequently telomerase activity, leading to premature telomeres shortening [82–84]. This inhibition has been shown in *in vitro* systems [83,84] but the role of NRTIs in telomerase activity and subsequent telomere length in HIV-infected patients is an update question. Cotè et al. investigated whether in utero or childhood exposure to NRTIs affects leukocyte telomere length (LTL) [85]. They studied LTL in 94 HIV-infected (HIV+) children, 177 HIV-exposed uninfected (HEU; born to HIV-infected mothers) children who were exposed to ART perinatally and 104 HIV-unexposed uninfected (HIV-) control children. It was observed that there was no difference in LTL between the HIV+, HEU and HIV- groups, so there were no associations between children’s LTL and their perinatal ART exposure or HIV infection; however, among HIV+ children an association was found between HIV load and LTL shortening [85]. In multivariate models older age (as expected) and male gender were the only factors associated with shorter LTL [85]. There is only one study in which both the immunosenescence profile and the leukocyte telomere length were analyzed in 0-5 years old age-matched groups of HIV+, HEU and HIV- children [86]. The percentages of senescent (CD28^-^CD57^+^), activated (CD38^+^HLADR^-^), and exhausted (PD1^+^) CD8 cells were significantly higher in HIV+ than in HEU and HIV- children, and LTL was significantly shorter in HIV+ than in HEU and HIV- groups, and, within the HIV+ group, in children without therapy. The different results of the two studies concerning the LTL marker may be caused by the different age of children enrolled in the two studies (0-5 vs 0-19 years old) [86]. Indeed, the telomere shortening is more rapid during the first years of life [87]. Thus, the difference between HIV-infected children and controls may emerge more clearly in a cohort of younger children. Finding that HIV-infected children accumulate CD8^+^CD38^+^ and CD8^+^PD1^+^ cells together with a higher percentage of senescent CD8^+^ cells is compatible with a scenario in which viremia leads to high turnover with continual loss and output of naive cells, which rapidly differentiate and exhaust their effector function, resulting in an accumulation of senescent cells with short telomeres. Furthermore, the finding that activated and exhausted CD8^+^ cells are inversely correlated with telomere length supports the idea that persistent immune activation and cellular exhaustion are closely linked to accelerated biological aging and immune senescence [86]. Chronic immune activation because of persistence of circulating virions may play a role in the senescence pathway; activated cells undergo clonal expansion in response to viral persistence, resulting in differentiation and accumulation of non functional senescent cells [88].

Moreover, it has been demonstrated that HIV infection *itself* and HIV Tat protein downmodulate telomerase expression and activity in lymphoblastoid cells and in peripheral blood cells lymphocytes [89–91]. Further studies are needed to clarify the link between HIV viremia and LTL and to determine whether short-term or long-term uncontrolled HIV viremia is involved and to define whether telomeres shortening is transient or permanent.

### *Inflammation and aging*

An important feature of aging and most of age-related diseases is chronic inflammation. There is a overwhelming evidence that a state of mild inflammation, revealed by increased levels of pro-inflammatory cytokines, such as IL-6, IL-10, is associated to and predictive of many aging phenotypes, including immunosenescence. An important source of chronic inflammation in HIV-infected individuals is provided by microbial translocation due to damage to intestinal mucosa caused by massive HIV-induced T-cell depletion in the gut [92]. Translocation of intestinal bacteria and bacterial products into the bloodstream can activate the immune system by binding to receptors involved in the host inflammatory response, such as Toll-like receptors (TLRs). TLRs are pattern recognition receptors which recognize structural components belonging to bacteria, fungi and viruses, known as "pathogen-associated molecular patterns" (PAMPs), and activate the innate immune response [93]. PAMPs include bacterial lipopolysaccharide (LPS), 16S ribosomal DNA (16S rDNA), and CpG DNA. A recent study demonstrated that high levels of PAMPs, generated by microbial translocation (sCD14 and LPS) are associated with the risk of NHL [94].

The loss of mucosal surface integrity in the gut, due to the massive depletion of CD4^+^ T cells, involves not only increased mucosal permeability and consequent microbial translocation, but also an increase in "damage-associated molecular patterns" (DAMPs), endogenous molecules released after cell death, such as mitochondrial DNA (mtDNA) [95], high mobility group 1 protein (HMGB1) [96] and defensins [97]. The binding of PAMP and DAMP ligands to the extra- or intra-cellular domain of TLRs initiates a complex-signal transduction cascade which, *via* the NF-κB pathway, ultimately leads to increased transcription of pro-inflammatory cytokines, such as IL-6 and TNF-α [98]. The findings that in HIV-infected children levels of sCD14 were correlated with percentages of activated CD8^+^ cells, and that HIV-infected children had higher levels of IL-6 and TNF-α than HEU and HIV- children, additionally support the concept that premature immunesenescence in HIV-infected children is mediated through immune system activation and chronic inflammation [86].

### *Epigenetic mechanisms and aging*

A recent study found a correlation between CpG DNA methylation signature in blood cells of HIV- infected patients and premature immunosenescence [99]. DNA methylation status studied in peripheral blood cells from 137 HIV-infected individuals under ART was compared with that observed in peripheral blood cells from 44 healthy controls. By analyzing a set of 26,927 age-associated methylation sites, the authors found increased methylation changes in HIV-infected patients beyond their chronological age, that suggested about a 5 years increase in aging compared to healthy controls; moreover, the premature immunosenescence equally occurred in HIV-infected patients ART-treated for less than 5 years and in those treated for more than 12 years, suggesting that the infection *per se,* rather than therapies, accelerates the aging process [99]. These data partly differ from those of a similar study conducted in HIV-infected adults that reported an acceleration of age-associated methylation pattern of about 14 years [100]; this difference may be probably due to different cohorts. However, not all cells have displayed the same premature aging, and this is one of the major limitations of these studies. Further studies are needed to determine if the methylation status of DNA can affect the immune response to HIV infection *or viceversa.*

## CONCLUSIONS

After the introduction of ART, HIV infection became a chronic disease, but HIV-infected children have not yet the same life expectancy of healthy children. The infection behaves differently in children and adults, with important clinical and immunological differences. ART and HIV infection coexist in children from birth, moving up immunosenescence and aging processes; these are more pronounced in children with detectable viremia, focusing attention on the need for early and long-standing control of HIV replication. The pathogenic mechanisms of immunosenescence are various, not completely identified and partly different from those described in adults. Senescent phenotype of T cells makes children more susceptible to infections and less responsive to vaccination. HIV-infected children and adolescents should be carefully monitored for the prompt detection and early treatment of noninfectious disorders related to premature aging. Notably, lipid and bone metabolism, cancers, cardiovascular, renal, and neurological systems must be carefully monitored adopting screening programs and preventive measures in high risk populations, such as children with detectable viremia or with CD4/CD8 ratio inversion, mainly due to increased levels of senescent and/or activated CD8^+^ lymphocytes. The datum that no premature aging effect has been described regarding several organ systems may depend on the fact that no investigation has been executed to date and further studies are needed before excluding premature senescence of these organs. Finally, further studies regarding the mechanisms involved in premature aging are needed to search for potential targets of treatment.

## METHODS

In order to perform a narrative review of the available literature, we searched PubMed, Medline, EMBASE and Cochrane databases from January 2004 through December 2017, using the following key words including: “aging”, “children”, “HIV”, “AIDS”, “immunosenescence”, “pathogenesis”, “clinical conditions”. Articles were limited to English language and full text availability, and they were excluded if they were redundant or not pertinent. References of all relevant articles were also evaluated and studies published previously than 2004 or in adults were cited if considered relevant (Appendix). Results were critically summarized in the two paragraphs considering: 1) clinical conditions related to premature aging in HIV-infected children, and 2) pathogenetic mechanisms of premature aging in HIV-infected children.

## Supplementary Material

Appendix

## References

[1] Wing EJ. HIV and aging. Int J Infect Dis. 2016; 53:61–68. 10.1016/j.ijid.2016.10.004 27756678

[2] IeDEA Pediatric Working Group. Taking a critical look at the UNAIDS global estimates on paediatric and adolescent HIV survival and death. J Int AIDS Soc. 2017; 20:21952. 10.7448/IAS.20.1.21952 28691433 PMC5515022

[3] Rickabaugh TM, Kilpatrick RD, Hultin LE, Hultin PM, Hausner MA, Sugar CA, Althoff KN, Margolick JB, Rinaldo CR, Detels R, Phair J, Effros RB, Jamieson BD. The dual impact of HIV-1 infection and aging on naïve CD4 T-cells: additive and distinct patterns of impairment. PLoS One. 2011; 6:e16459. 10.1371/journal.pone.0016459 21298072 PMC3027697

[4] Kennedy BK, Berger SL, Brunet A, Campisi J, Cuervo AM, Epel ES, Franceschi C, Lithgow GJ, Morimoto RI, Pessin JE, Rando TA, Richardson A, Schadt EE, et al. Geroscience: linking aging to chronic disease. Cell. 2014; 159:709–13. 10.1016/j.cell.2014.10.039 25417146 PMC4852871

[5] Lagathu C, Cossarizza A, Béréziat V, Nasi M, Capeau J, Pinti M. Basic science and pathogenesis of ageing with HIV: potential mechanisms and biomarkers. AIDS. 2017 (Suppl 2); 31:S105–19. 10.1097/QAD.0000000000001441 28471941

[6] UNAIDS. UNAIDS. Gap report. [Internet]. Geneva, Switzerland: UNAIDS, 2014. Available from: http://www.unaids.org/sites/default/files/en/media/ unaids/contentassets/documents/unaidspublication/2014/ UNAIDS_Gap_report_en.pdf

[7] Deeks SG, Phillips AN. HIV infection, antiretroviral treatment, ageing, and non-AIDS related morbidity. BMJ. 2009; 338:a3172. 10.1136/bmj.a3172 19171560

[8] Hazra R, Siberry GK, Mofenson LM. Growing up with HIV: children, adolescents, and young adults with perinatally acquired HIV infection. Annu Rev Med. 2010; 61:169–85. 10.1146/annurev.med.050108.151127 19622036

[9] Brady MT, Oleske JM, Williams PL, Elgie C, Mofenson LM, Dankner WM, Van Dyke RB, and Pediatric AIDS Clinical Trials Group219/219C Team. Declines in mortality rates and changes in causes of death in HIV-1-infected children during the HAART era. J Acquir Immune Defic Syndr. 2010; 53:86–94. 10.1097/QAI.0b013e3181b9869f 20035164 PMC2801894

[10] Chiappini E, Berti E, Gianesin K, Petrara MR, Galli L, Giaquinto C, de Martino M, De Rossi A. Pediatric human immunodeficiency virus infection and cancer in the highly active antiretroviral treatment (HAART) era. Cancer Lett. 2014; 347:38–45. 10.1016/j.canlet.2014.02.002 24513180

[11] Guaraldi G, Palella FJ Jr. Clinical implications of aging with HIV infection: perspectives and the future medical care agenda. AIDS. 2017 (Suppl 2); 31:S129–35. 10.1097/QAD.0000000000001478 28471943

[12] De Rossi A, Masiero S, Giaquinto C, Ruga E, Comar M, Giacca M, Chieco-Bianchi L. Dynamics of viral replication in infants with vertically acquired human immunodeficiency virus type 1 infection. J Clin Invest. 1996; 97:323–30. 10.1172/JCI118419 8567951 PMC507021

[13] Prendergast AJ, Klenerman P, Goulder PJ. The impact of differential antiviral immunity in children and adults. Nat Rev Immunol. 2012; 12:636–48. 10.1038/nri3277 22918466

[14] Sollai S, Noguera-Julian A, Galli L, Fortuny C, Deyà Á, de Martino M, Chiappini E. Strategies for the prevention of mother to child transmission in Western countries: an update. Pediatr Infect Dis J. 2015 (Suppl 1); 34:S14–30. 10.1097/INF.0000000000000661 25894973

[15] Judd A, Doerholt K, Tookey PA, Sharland M, Riordan A, Menson E, Novelli V, Lyall EG, Masters J, Tudor-Williams G, Duong T, Gibb DM, and Collaborative HIV Paediatric Study (CHIPS), and National Study of HIV in Pregnancy and Childhood (NSHPC). Morbidity, mortality, and response to treatment by children in the United Kingdom and Ireland with perinatally acquired HIV infection during 1996-2006: planning for teenage and adult care. Clin Infect Dis. 2007; 45:918–24. 10.1086/521167 17806062

[16] Cohort Collaboration (EPPICC) study group in EuroCoord. Judd A, Chappell E, Turkova A, Le Coeur S, Noguera-Julian A, Goetghebuer T, Doerholt K, Galli L, Pajkrt D, Marques L, Collins IJ, Gibb DM, González Tome MI, Navarro M, Warszawski J, Königs C, Spoulou V, Prata F, Chiappini E, Naver L, Giaquinto C, Thorne C, Marczynska M, Okhonskaia L, Posfay-Barbe K, Ounchanum P, Techakunakorn P, Kiseleva G, Malyuta R, Volokha A, Ene L, Goodall R. Long-term trends in mortality and AIDS-defining events after combination ART initiation among children and adolescents with perinatal HIV infection in 17 middle- and high-income countries in Europe and Thailand: A cohort study. European Pregnancy and Paediatric HIV. PLoS Med. 2018; 15:e100249. 10.1371/journal.pmed.1002491 PMC5790238 29381702

[17] McCulloch MI, Ray PE. Kidney disease in HIV-positive children. Semin Nephrol. 2008; 28:585–94. 10.1016/j.semnephrol.2008.09.001 19013330 PMC2778302

[18] Rakhmanina N, Wong EC, Davis JC, Ray PE. Hemorrhagic Stroke in an Adolescent Female with HIV-Associated Thrombotic Thrombocytopenic Purpura. J AIDS Clin Res. 2014; 5:311. 10.4172/2155-6113.1000311 25429351 PMC4241775

[19] Barrios A, García-Benayas T, González-Lahoz J, Soriano V. Tenofovir-related nephrotoxicity in HIV-infected patients. AIDS. 2004; 18:960–63. 10.1097/00002030-200404090-00019 15060449

[20] Perazzo S, Soler-García ÁA, Hathout Y, Das JR, Ray PE. Urinary biomarkers of kidney diseases in HIV-infected children. Proteomics Clin Appl. 2015; 9:490–500. 10.1002/prca.201400193 25764519 PMC4530778

[21] Deyà-Martínez À, Fortuny C, Soler-Palacín P, Neth O, Sánchez E, Martín-Nalda A, Falcón-Neyra L, Vila A, Valls A, Noguera-Julian A, Cystatin C. Cystatin C: A Marker for Inflammation and Renal Function Among HIV-infected Children and Adolescents. Pediatr Infect Dis J. 2016; 35:196–200. 10.1097/INF.0000000000000960 26479972

[22] Bhimma R, Purswani MU, Kala U. Kidney disease in children and adolescents with perinatal HIV-1 infection. J Int AIDS Soc. 2013; 16:18596. 10.7448/IAS.16.1.18596 23782479 PMC3687339

[23] Purswani MU, Patel K, Winkler CA, Spector SA, Hazra R, Seage GR 3rd, Mofenson L, Karalius B, Scott GB, Van Dyke RB, Kopp JB, and Pediatric HIVAIDS Cohort Study. Brief Report: APOL1 Renal Risk Variants Are Associated With Chronic Kidney Disease in Children and Youth With Perinatal HIV Infection. J Acquir Immune Defic Syndr. 2016; 73:63–68. 10.1097/QAI.0000000000001010 27035887 PMC4981510

[24] Das JR, Gutkind JS, Ray PE. Circulating Fibroblast Growth Factor-2, HIV-Tat, and vascular endothelial cell growth factor-A in HIV-Infected children with renal disease activate Rho-A and Src in cultured renal endothelial cells. PLoS One. 2016; 11:e0153837. 10.1371/journal.pone.0153837 27097314 PMC4838216

[25] Li J, Das JR, Tang P, Han Z, Jaiswal JK, Ray PE. Transmembrane TNF-*α* Facilitates HIV-1 Infection of Podocytes Cultured from Children with HIV-Associated Nephropathy. J Am Soc Nephrol. 2017; 28:862–75. 10.1681/ASN.2016050564 27811066 PMC5328167

[26] Cooper ER, Hanson C, Diaz C, Mendez H, Abboud R, Nugent R, Pitt J, Rich K, Rodriguez EM, Smeriglio V, and Women and Infants Transmission Study Group. Encephalopathy and progression of human immunodeficiency virus disease in a cohort of children with perinatally acquired human immunodeficiency virus infection. J Pediatr. 1998; 132:808–12. 10.1016/S0022-3476(98)70308-7 9602190

[27] Patel K, Ming X, Williams PL, Robertson KR, Oleske JM, Seage GR 3rd, and International Maternal Pediatric Adolescent AIDS Clinical Trials 219/219C Study Team. Impact of HAART and CNS-penetrating antiretroviral regimens on HIV encephalopathy among perinatally infected children and adolescents. AIDS. 2009; 23:1893–901. 10.1097/QAD.0b013e32832dc041 19644348 PMC2821205

[28] Puthanakit T, Ananworanich J, Vonthanak S, Kosalaraksa P, Hansudewechakul R, van der Lugt J, Kerr SJ, Kanjanavanit S, Ngampiyaskul C, Wongsawat J, Luesomboon W, Vibol U, Pruksakaew K, et al, and PREDICT Study Group. Cognitive function and neurodevelopmental outcomes in HIV-infected Children older than 1 year of age randomized to early versus deferred antiretroviral therapy: the PREDICT neurodevelopmental study. Pediatr Infect Dis J. 2013; 32:501–08. 10.1097/INF.0b013e31827fb19d 23263176 PMC3664246

[29] Cohen S, Ter Stege JA, Geurtsen GJ, Scherpbier HJ, Kuijpers TW, Reiss P, Schmand B, Pajkrt D. Poorer cognitive performance in perinatally HIV-infected children versus healthy socioeconomically matched controls. Clin Infect Dis. 2015; 60:1111–19. 10.1093/cid/ciu1144 25516183

[30] Vreeman RC, Scanlon ML, McHenry MS, Nyandiko WM. The physical and psychological effects of HIV infection and its treatment on perinatally HIV-infected children. J Int AIDS Soc. 2015 (Suppl 6); 18:20258. 10.7448/IAS.18.7.20258 26639114 PMC4670835

[31] Wilmshurst JM, Hammond CK, Donald K, Hoare J, Cohen K, Eley B. NeuroAIDS in children. Handb Clin Neurol. 2018; 152:99–116. 10.1016/B978-0-444-63849-6.00008-6 29604987

[32] van Arnhem LA, Bunders MJ, Scherpbier HJ, Majoie CB, Reneman L, Frinking O, Poll-The BT, Kuijpers TW, Pajkrt D. Neurologic abnormalities in HIV-1 infected children in the era of combination antiretroviral therapy. PLoS One. 2013; 8:e64398. 10.1371/journal.pone.0064398 23691211 PMC3654960

[33] Ackermann C, Andronikou S, Laughton B, Kidd M, Dobbels E, Innes S, van Toorn R, Cotton M. White matter signal abnormalities in children with suspected HIV-related neurologic disease on early combination antiretroviral therapy. Pediatr Infect Dis J. 2014; 33:e207–12. 10.1097/INF.0000000000000288 24595047 PMC4153800

[34] Connor MD. Treatment of HIV associated cerebral vasculopathy. J Neurol Neurosurg Psychiatry. 2009; 80:831. 10.1136/jnnp.2008.169490 19608782

[35] Hammond CK, Eley B, Wieselthaler N, Ndondo A, Wilmshurst JM. Cerebrovascular disease in children with HIV-1 infection. Dev Med Child Neurol. 2016; 58:452–60. 10.1111/dmcn.13080 26890389

[36] Eckard AR, Mora S. Bone health in HIV-infected children and adolescents. Curr Opin HIV AIDS. 2016; 11:294–300. 10.1097/COH.0000000000000270 26890208 PMC4860809

[37] Palchetti CZ, Szejnfeld VL, de Menezes Succi RC, Patin RV, Teixeira PF, Machado DM, Oliveira FL. Impaired bone mineral accrual in prepubertal HIV-infected children: a cohort study. Braz J Infect Dis. 2015; 19:623–30. 10.1016/j.bjid.2015.08.010 26477385 PMC9425359

[38] Warriner AH, Mugavero M, Overton ET. Bone alterations associated with HIV. Curr HIV/AIDS Rep. 2014; 11:233–40. 10.1007/s11904-014-0216-x 25064454

[39] Mansky KC. Aging, human immunodeficiency virus, and bone health. Clin Interv Aging. 2010; 5:285–92. 10.2147/CIA.S13852 20924437 PMC2946855

[40] Puthanakit T, Siberry GK. Bone health in children and adolescents with perinatal HIV infection. J Int AIDS Soc. 2013; 16:18575. 10.7448/IAS.16.1.18575 23782476 PMC3687077

[41] Natsag J, Kendall MA, Sellmeyer DE, McComsey GA, Brown TT. Vitamin D, osteoprotegerin/receptor activator of nuclear factor-kappaB ligand (OPG/RANKL) and inflammation with alendronate treatment in HIV-infected patients with reduced bone mineral density. HIV Med. 2016; 17:196–205. 10.1111/hiv.12291 26177791 PMC4715784

[42] Gibellini D, De Crignis E, Ponti C, Cimatti L, Borderi M, Tschon M, Giardino R, Re MC. HIV-1 triggers apoptosis in primary osteoblasts and HOBIT cells through TNFalpha activation. J Med Virol. 2008; 80:1507–14. 10.1002/jmv.21266 18649336

[43] Beaupere C, Garcia M, Larghero J, Fève B, Capeau J, Lagathu C. The HIV proteins Tat and Nef promote human bone marrow mesenchymal stem cell senescence and alter osteoblastic differentiation. Aging Cell. 2015; 14:534–46. 10.1111/acel.12308 25847297 PMC4531068

[44] Manavalan JS, Arpadi S, Tharmarajah S, Shah J, Zhang CA, Foca M, Neu N, Bell DL, Nishiyama KK, Kousteni S, Yin MT. Abnormal Bone Acquisition With Early-Life HIV Infection: Role of Immune Activation and Senescent Osteogenic Precursors. J Bone Miner Res. 2016; 31:1988–96. 10.1002/jbmr.2883 27283956 PMC5399769

[45] Jiménez B, Sainz T, Díaz L. avarro ML, Rojo P, González-Tomé MI, Prieto L, Martínez J, de José MI, Ramos JT, Muñoz-Fernandez MÁ; Madrid Cohort of HIV-Infected Children and Adolescents Integrated in the Pediatric Branch of the Spanish National AIDS Research Network (CoRISpe). Low Bone Mineral Density in Vertically HIV-infected Children and Adolescents. Pediatr Infect Dis J. 2017; 36:578–83. 10.1097/INF.0000000000001506 28005690

[46] Idris NS, Grobbee DE, Burgner D, Cheung MM, Kurniati N, Sastroasmoro S, Uiterwaal CS. Cardiovascular manifestations of HIV infection in children. Eur J Prev Cardiol. 2015; 22:1452–61. 10.1177/2047487314560086 25398702

[47] Lorenz MW, Stephan C, Harmjanz A, Staszewski S, Buehler A, Bickel M, von Kegler S, Ruhkamp D, Steinmetz H, Sitzer M. Both long-term HIV infection and highly active antiretroviral therapy are independent risk factors for early carotid atherosclerosis. Atherosclerosis. 2008; 196:720–26. 10.1016/j.atherosclerosis.2006.12.022 17275008

[48] Kaplan RC, Sinclair E, Landay AL, Lurain N, Sharrett AR, Gange SJ, Xue X, Hunt P, Karim R, Kern DM, Hodis HN, Deeks SG. T cell activation and senescence predict subclinical carotid artery disease in HIV-infected women. J Infect Dis. 2011; 203:452–63. 10.1093/infdis/jiq071 21220772 PMC3071219

[49] Di Biagio A, Rosso R, Maggi P, Mazzei D, Bernardini C, Nulvesu L, Parisini A, Nicco E, De Carli F, Rodriguez G, Viscoli C. Inflammation markers correlate with common carotid intima-media thickness in patients perinatally infected with human immunodeficiency virus 1. J Ultrasound Med. 2013; 32:763–68. 10.7863/ultra.32.5.763 23620317

[50] Sainz T, Álvarez-Fuente M, Navarro ML, Díaz L, Rojo P, Blázquez D, de José MI, Ramos JT, Serrano-Villar S, Martínez J, Medrano C, Muñoz-Fernández MÁ, Mellado MJ. Madrid Cohort of HIV-infected children and adolescents integrated in the Pediatric branch of the Spanish National AIDS Network (CoRISPE). Subclinical Atherosclerosis and Markers of Immune Activation in HIV-Infected Children and Adolescents. J Acquir Immune Defic Syndr. 2014; 65:42–49. 10.1097/QAI.0b013e3182a9466a 23982657

[51] Mattingly AS, Unsal AB, Purdy JB, Gharib AM, Rupert A, Kovacs JA, McAreavey D, Hazra R, Abd-Elmoniem KZ, Hadigan C. T-cell Activation and E-selectin Are Associated With Coronary Plaque in HIV-infected Young Adults. Pediatr Infect Dis J. 2017; 36:63–65. 10.1097/INF.0000000000001354 27749650 PMC5154863

[52] Ross AC, O’Riordan MA, Storer N, Dogra V, McComsey GA. Heightened inflammation is linked to carotid intima-media thickness and endothelial activation in HIV-infected children. Atherosclerosis. 2010; 211:492–98. 10.1016/j.atherosclerosis.2010.04.008 20471650

[53] Loomba-Albrecht LA, Bregman T, Chantry CJ. Endocrinopathies in children infected with human immunodeficiency virus. Endocrinol Metab Clin North Am. 2014; 43:807–28. 10.1016/j.ecl.2014.06.001 25169569

[54] Chiappini E, Galli L, Tovo PA, Gabiano C, Lisi C, Giaquinto C, Rampon O, Gattinara GC, De Marco G, Osimani P, Manzionna M, Miniaci A, Pintor C, et al, and Italian Register for HIV Infection in Children. Cancer rates after year 2000 significantly decrease in children with perinatal HIV infection: a study by the Italian Register for HIV Infection in Children. J Clin Oncol. 2007; 25:97–101. 10.1200/JCO.2006.06.6506 17194910

[55] Alvaro-Meca A, Micheloud D, Jensen J, Díaz A, García-Alvarez M, Resino S. Epidemiologic trends of cancer diagnoses among HIV-infected children in Spain from 1997 to 2008. Pediatr Infect Dis J. 2011; 30:764–68. 10.1097/INF.0b013e31821ba148 21494172

[56] Davidson A, Wainwright RD, Stones DK, Kruger M, Hendricks M, Geel J, Poole J, Reynders D, Omar F, Mathew R, Stefan DC. Malignancies in South African children with HIV. J Pediatr Hematol Oncol. 2014; 36:111–17. 10.1097/MPH.0b013e31829cdd49 24552745

[57] Simard EP, Shiels MS, Bhatia K, Engels EA. Long-term cancer risk among people diagnosed with AIDS during childhood. Cancer Epidemiol Biomarkers Prev. 2012; 21:148–54. 10.1158/1055-9965.EPI-11-0823 22068287 PMC3312034

[58] Franceschi S, Lise M, Clifford GM, Rickenbach M, Levi F, Maspoli M, Bouchardy C, Dehler S, Jundt G, Ess S, Bordoni A, Konzelmann I, Frick H, et al, and Swiss HIV Cohort Study. Changing patterns of cancer incidence in the early- and late-HAART periods: the Swiss HIV Cohort Study. Br J Cancer. 2010; 103:416–22. 10.1038/sj.bjc.6605756 20588274 PMC2920013

[59] Bains I, Thiébaut R, Yates AJ, Callard R. Quantifying thymic export: combining models of naive T cell proliferation and TCR excision circle dynamics gives an explicit measure of thymic output. J Immunol. 2009; 183:4329–36. 10.4049/jimmunol.0900743 19734223

[60] Franceschi C, Campisi J. Chronic inflammation (inflammaging) and its potential contribution to age-associated diseases. J Gerontol A Biol Sci Med Sci. 2014 (Suppl 1); 69:S4–9. 10.1093/gerona/glu057 24833586

[61] Mansoor N, Abel B, Scriba TJ, Hughes J, de Kock M, Tameris M, Mlenjeni S, Denation L, Little F, Gelderbloem S, Hawkridge A, Boom WH, Kaplan G, et al. Significantly skewed memory CD8+ T cell subsets in HIV-1 infected infants during the first year of life. Clin Immunol. 2009; 130:280–89. 10.1016/j.clim.2008.09.006 18996749 PMC2722743

[62] Sallusto F, Geginat J, Lanzavecchia A. Central memory and effector memory T cell subsets: function, generation, and maintenance. Annu Rev Immunol. 2004; 22:745–63. 10.1146/annurev.immunol.22.012703.104702 15032595

[63] Brenchley JM, Karandikar NJ, Betts MR, Ambrozak DR, Hill BJ, Crotty LE, Casazza JP, Kuruppu J, Migueles SA, Connors M, Roederer M, Douek DC, Koup RA. Expression of CD57 defines replicative senescence and antigen-induced apoptotic death of CD8+ T cells. Blood. 2003; 101:2711–20. 10.1182/blood-2002-07-2103 12433688

[64] Díaz L, Méndez-Lagares G, Correa-Rocha R, Pacheco YM, Ferrando-Martínez S, Ruiz-Mateos E, del Mar del Pozo-Balado M, León JA, Gurbindo MD, Isabel de José M, Leal M, Muñoz-Fernández MÁ. Detectable viral load aggravates immunosenescence features of CD8 T-cell subsets in vertically HIV-infected children. J Acquir Immune Defic Syndr. 2012; 60:447–54. 10.1097/QAI.0b013e318259254f 22549383

[65] Effros RB. Replicative senescence of CD8 T cells: potential effects on cancer immune surveillance and immunotherapy. Cancer Immunol Immunother. 2004; 53:925–33. 10.1007/s00262-004-0508-x 15067431 PMC11032951

[66] Campisi J, Andersen JK, Kapahi P, Melov S. Cellular senescence: a link between cancer and age-related degenerative disease? Semin Cancer Biol. 2011; 21:354–59. 10.1016/j.semcancer.2011.09.001 21925603 PMC3230665

[67] Sainz T, Serrano-Villar S, Díaz L, González Tomé MI, Gurbindo MD, de José MI, Mellado MJ, Ramos JT, Zamora J, Moreno S, Muñoz-Fernández MA. The CD4/CD8 ratio as a marker T-cell activation, senescence and activation/exhaustion in treated HIV-infected children and young adults. AIDS. 2013; 27:1513–16. 10.1097/QAD.0b013e32835faa72 23435292

[68] Viganò A, Pinti M, Nasi M, Moretti L, Balli F, Mussini C, Bricalli D, Sala N, Bugarini R, Vella S, Principi N, Cossarizza A. Markers of cell death-activation in lymphocytes of vertically HIV-infected children naive to highly active antiretroviral therapy: the role of age. J Allergy Clin Immunol. 2001; 108:439–45. 10.1067/mai.2001.117791 11544465

[69] Hart M, Steel A, Clark SA, Moyle G, Nelson M, Henderson DC, Wilson R, Gotch F, Gazzard B, Kelleher P. Loss of discrete memory B cell subsets is associated with impaired immunization responses in HIV-1 infection and may be a risk factor for invasive pneumococcal disease. J Immunol. 2007; 178:8212–20. 10.4049/jimmunol.178.12.8212 17548660

[70] Siberry GK, Patel K, Bellini WJ, Karalius B, Purswani MU, Burchett SK, Meyer WA 3rd, Sowers SB, Ellis A, Van Dyke RB, Pediatric HI, Pediatric HI, and Pediatric HIV AIDS Cohort Study (PHACS), and Pediatric HIV AIDS Cohort Study PHACS. Immunity to Measles, Mumps, and Rubella in US Children With Perinatal HIV Infection or Perinatal HIV Exposure Without Infection. Clin Infect Dis. 2015; 61:988–95. 10.1093/cid/civ440 26060291 PMC4551008

[71] Cagigi A, Rinaldi S, Di Martino A, Manno EC, Zangari P, Aquilani A, Cotugno N, Nicolosi L, Villani A, Bernardi S, Donatelli I, Pahwa S, Rossi P, Palma P. Premature immune senescence during HIV-1 vertical infection relates with response to influenza vaccination. J Allergy Clin Immunol. 2014; 133:592–94. 10.1016/j.jaci.2013.10.003 24290278

[72] Moir S, Ho J, Malaspina A, Wang W, DiPoto AC, O’Shea MA, Roby G, Kottilil S, Arthos J, Proschan MA, Chun TW, Fauci AS. Evidence for HIV-associated B cell exhaustion in a dysfunctional memory B cell compartment in HIV-infected viremic individuals. J Exp Med. 2008; 205:1797–805. 10.1084/jem.20072683 18625747 PMC2525604

[73] Moir S, Fauci AS. B cells in HIV infection and disease. Nat Rev Immunol. 2009; 9:235–45. 10.1038/nri2524 19319142 PMC2779527

[74] Rinaldi S, Pallikkuth S, George VK, de Armas LR, Pahwa R, Sanchez CM, Pallin MF, Pan L, Cotugno N, Dickinson G, Rodriguez A, Fischl M, Alcaide M, et al. Paradoxical aging in HIV: immune senescence of B Cells is most prominent in young age. Aging (Albany NY). 2017; 9:1307–25. 10.18632/aging.101229 28448963 PMC5425129

[75] Giunco S, Celeghin A, Gianesin K, Dolcetti R, Indraccolo S, De Rossi A. Cross talk between EBV and telomerase: the role of TERT and NOTCH2 in the switch of latent/lytic cycle of the virus. Cell Death Dis. 2015; 6:e1774. 10.1038/cddis.2015.145 26018735 PMC4669716

[76] Tchkonia T, Zhu Y, van Deursen J, Campisi J, Kirkland JL. Cellular senescence and the senescent secretory phenotype: therapeutic opportunities. J Clin Invest. 2013; 123:966–72. 10.1172/JCI64098 23454759 PMC3582125

[77] Shay JW. Telomeres and aging. Curr Opin Cell Biol. 2018; 52:1–7. 10.1016/j.ceb.2017.12.001 29253739

[78] De Meyer T, Rietzschel ER, De Buyzere ML, Van Criekinge W, Bekaert S. Telomere length and cardiovascular aging: the means to the ends? Ageing Res Rev. 2011; 10:297–303. 10.1016/j.arr.2010.11.001 21109027

[79] Artandi SE, DePinho RA. Telomeres and telomerase in cancer. Carcinogenesis. 2010; 31:9–18. 10.1093/carcin/bgp268 19887512 PMC3003493

[80] Gillis AJ, Schuller AP, Skordalakes E. Structure of the Tribolium castaneum telomerase catalytic subunit TERT. Nature. 2008; 455:633–37. 10.1038/nature07283 18758444

[81] Peng Y, Mian IS, Lue NF. Analysis of telomerase processivity: mechanistic similarity to HIV-1 reverse transcriptase and role in telomere maintenance. Mol Cell. 2001; 7:1201–11. 10.1016/S1097-2765(01)00268-4 11430823

[82] Tressler R, Godfrey C. NRTI backbone in HIV treatment: will it remain relevant? Drugs. 2012; 72:2051–62. 10.2165/11640830-000000000-00000 23083109

[83] Liu X, Takahashi H, Harada Y, Ogawara T, Ogimura Y, Mizushina Y, Saneyoshi M, Yamaguchi T. 3′-Azido-2′,3′-dideoxynucleoside 5′-triphosphates inhibit telomerase activity in vitro, and the corresponding nucleosides cause telomere shortening in human HL60 cells. Nucleic Acids Res. 2007; 35:7140–49. 10.1093/nar/gkm859 17942424 PMC2175342

[84] Hukezalie KR, Thumati NR, Côté HC, Wong JM. In vitro and ex vivo inhibition of human telomerase by anti-HIV nucleoside reverse transcriptase inhibitors (NRTIs) but not by non-NRTIs. PLoS One. 2012; 7:e47505. 10.1371/journal.pone.0047505 23166583 PMC3499584

[85] Côté HC, Soudeyns H, Thorne A, Alimenti A, Lamarre V, Maan EJ, Sattha B, Singer J, Lapointe N, Money DM, Forbes J, Wong J, Bitnun A, et al, and CIHR Emerging Team in HIV therapy, aging (CARMA). Leukocyte telomere length in HIV-infected and HIV-exposed uninfected children: shorter telomeres with uncontrolled HIV viremia. PLoS One. 2012; 7:e39266. 10.1371/journal.pone.0039266 22815702 PMC3397986

[86] Gianesin K, Noguera-Julian A, Zanchetta M, Del Bianco P, Petrara MR, Freguja R, Rampon O, Fortuny C, Camós M, Mozzo E, Giaquinto C, De Rossi A. Premature aging and immune senescence in HIV-infected children. AIDS. 2016; 30:1363–73. 10.1097/QAD.0000000000001093 26990630 PMC4867984

[87] Zeichner SL, Palumbo P, Feng Y, Xiao X, Gee D, Sleasman J, Goodenow M, Biggar R, Dimitrov D. Rapid telomere shortening in children. Blood. 1999; 93:2824–30. 10216076

[88] Desai S, Landay A. Early immune senescence in HIV disease. Curr HIV/AIDS Rep. 2010; 7:4–10. 10.1007/s11904-009-0038-4 20425052 PMC3739442

[89] Ballon G, Ometto L, Righetti E, Cattelan AM, Masiero S, Zanchetta M, Chieco-Bianchi L, De Rossi A, and De Rossi A. Human immunodeficiency virus type 1 modulates telomerase activity in peripheral blood lymphocytes. J Infect Dis. 2001; 183:417–24. 10.1086/318072 11133373

[90] Reynoso R, Minces L, Salomon H, Quarleri J. HIV-1 infection downregulates nuclear telomerase activity on lymphoblastoic cells without affecting the enzymatic components at the transcriptional level. AIDS Res Hum Retroviruses. 2006; 22:425–29. 10.1089/aid.2006.22.425 16706619

[91] Franzese O, Adamo R, Pollicita M, Comandini A, Laudisi A, Perno CF, Aquaro S, Bonmassar E. Telomerase activity, hTERT expression, and phosphorylation are downregulated in CD4(+) T lymphocytes infected with human immunodeficiency virus type 1 (HIV-1). J Med Virol. 2007; 79:639–46. 10.1002/jmv.20855 17387751

[92] Brenchley JM, Price DA, Schacker TW, Asher TE, Silvestri G, Rao S, Kazzaz Z, Bornstein E, Lambotte O, Altmann D, Blazar BR, Rodriguez B, Teixeira-Johnson L, et al. Microbial translocation is a cause of systemic immune activation in chronic HIV infection. Nat Med. 2006; 12:1365–71. 10.1038/nm1511 17115046

[93] Janeway CA Jr, Medzhitov R. Innate immune recognition. Annu Rev Immunol. 2002; 20:197–216. 10.1146/annurev.immunol.20.083001.084359 11861602

[94] Marks MA, Rabkin CS, Engels EA, Busch E, Kopp W, Rager H, Goedert JJ, Chaturvedi AK. Markers of microbial translocation and risk of AIDS-related lymphoma. AIDS. 2013; 27:469–74. 10.1097/QAD.0b013e32835c1333 23169327

[95] Zhang Q, Raoof M, Chen Y, Sumi Y, Sursal T, Junger W, Brohi K, Itagaki K, Hauser CJ. Circulating mitochondrial DAMPs cause inflammatory responses to injury. Nature. 2010; 464:104–07. 10.1038/nature08780 20203610 PMC2843437

[96] Scaffidi P, Misteli T, Bianchi ME. Release of chromatin protein HMGB1 by necrotic cells triggers inflammation. Nature. 2002; 418:191–95. 10.1038/nature00858 12110890

[97] Sørensen OE, Thapa DR, Rosenthal A, Liu L, Roberts AA, Ganz T. Differential regulation of beta-defensin expression in human skin by microbial stimuli. J Immunol. 2005; 174:4870–79. 10.4049/jimmunol.174.8.4870 15814714

[98] Petrara MR, Freguja R, Gianesin K, Zanchetta M, De Rossi A. Epstein-Barr virus-driven lymphomagenesis in the context of human immunodeficiency virus type 1 infection. Front Microbiol. 2013; 4:311. 10.3389/fmicb.2013.00311 24151490 PMC3799006

[99] Gross AM, Jaeger PA, Kreisberg JF, Licon K, Jepsen KL, Khosroheidari M, Morsey BM, Swindells S, Shen H, Ng CT, Flagg K, Chen D, Zhang K, et al. Methylome-wide Analysis of Chronic HIV Infection Reveals Five-Year Increase in Biological Age and Epigenetic Targeting of HLA. Mol Cell. 2016; 62:157–68. 10.1016/j.molcel.2016.03.019 27105112 PMC4995115

[100] Rickabaugh TM, Baxter RM, Sehl M, Sinsheimer JS, Hultin PM, Hultin LE, Quach A, Martínez-Maza O, Horvath S, Vilain E, Jamieson BD. Acceleration of age-associated methylation patterns in HIV-1-infected adults. PLoS One. 2015; 10:e0119201. 10.1371/journal.pone.0119201 25807146 PMC4373843

