# Supplementary material for: Accelerated aging in perinatally HIV-infected children: clinical manifestations and pathogenetic mechanisms
Source: Aging (Albany NY). 2018 Nov 11;10(11):3610–25. doi: 10.18632/aging.101622 (PMC6286860; doi:10.18632/aging.101622)
Supplement: Appendix [file aging-10-101622-s001.pdf]

APPENDIX

1) Search strategy for pathogenetic mechanisms in Medline/PubMed: ”(Children[Title/Abstract] AND (HIV[Title/Abstract] OR (immunodeficiency[Title/Abstract] AND virus[Title/Abstract]) OR (antiretroviral [Title/Abstract] AND therapy[Title/Abstract]) OR ART[Title/Abstract] OR haart[Title/Abstract]) AND (pathogenesis[Title/Abstract] OR aging[Title/Abstract] OR senescence[Title/Abstract] OR senescent[Title/Abstract] OR apoptosis[Title/Abstract] OR (chronic[Title/Abstract] AND inflammation[Title/Abstract]) OR telomeres[Title/Abstract] OR epigenetic[Title/Abstract] OR (immune[Title/Abstract] AND activation[Title/Abstract]) OR exhaustion[Title/Abstract]) AND (("2004/01/01"[PDAT] : "2017/12/31"[PDAT]) AND "humans"[MeSH Terms] AND English[lang])

Results: 365 articles were initially retrieved and additional 35 studies where recovered from references of selected studies; 360 articles were excluded because not pertinent or related to adults; finally 40 articles where selected for the present narrative review.

2) Search strategy for clinical conditions related to premature aging in Medline/PubMed:” Children[Title/Abstract] AND (HIV[Title/Abstract] OR (immunodeficiency[Title/Abstract] AND virus[Title/Abstract]) OR (antiretroviral [Title/Abstract] AND therapy[Title/Abstract]) OR ART[Title/Abstract] OR haart[Title/Abstract]) AND (pathogenesis[Title/Abstract] OR aging[Title/Abstract] OR senescence[Title/Abstract] OR senescent[Title/Abstract] OR apoptosis[Title/Abstract]) AND (cardiovascular[Title/Abstract] OR (intima[Title/Abstract] AND thickness [Title/Abstract]) OR pressure[Title/Abstract] OR heart[Title/Abstract] OR vasculitis[Title/Abstract] OR (organ [Title/Abstract] AND failure[Title/Abstract]) OR (organ[Title/Abstract] AND damage[Title/Abstract]) OR kidney[Title/Abstract] OR liver[Title/Abstract] OR renal[Title/Abstract] OR nephropathy[Title/Abstract] OR nephro-logic[Title/Abstract] OR cardiologic[Title/Abstract] OR neurological[Title/Abstract] OR psycological[Title/Abstract] OR bone[Title/Abstract] OR hormone[Title/Abstract] OR endocrinological[Title/Abstract] OR glucose[Title/Abstract] OR lipid[Title/Abstract] OR lypodystrophy[Title/Abstract] OR cancer[Title/Abstract] OR lymphoma[Title/Abstract] OR skin[Title/Abstract] OR cutaneous[Title/Abstract] OR muscle[Title/Abstract] OR lung[Title/Abstract] OR pneumologic[Title/Abstract] OR genital[Title/Abstract] OR fertility[Title/Abstract] OR encephalopathy[Title/Abstract] OR incidence[Title/Abstract] OR (adverse[Title/Abstract] AND event[Title/Abstract])) AND (("2004/01/01"[PDAT] : "2017/12/31"[PDAT]) AND "humans"[MeSH Terms] AND English[lang]) AND (("2004/01/01"[PDAT] : "2017/12/31"[PDAT]) AND "humans"[MeSH Terms] AND English[lang])

Results: 91 articles were initially retrieved and additional 12 studies where recovered from references; 66 articles were excluded because not pertinent or related to adults; finally 37 where selected for the present narrative review.
